# Stress-associated weight gain, fibromyalgia symptoms, cardiometabolic markers, and human growth hormone suppression respond to an amino acid supplement blend: Results of a prospective, cohort study

**DOI:** 10.3389/fendo.2023.1053692

**Published:** 2023-03-14

**Authors:** Susan Pekarovics, Adam Beres, Colleen Kelly, Sonja K. Billes, Amy L. Heaton

**Affiliations:** ^1^ Susan Pekarovics, MD, Professional Medical Corporation, Los Angeles, CA, United States; ^2^ Attending Physician, Cedars-Sinai Medical Center, Los Angeles, CA, United States; ^3^ Kelly Statistical Consulting, San Diego, CA, United States; ^4^ August Scientific, Encinitas, CA, United States; ^5^ Sierra Research Group, LLC, Salt Lake City, UT, United States; ^6^ Pennington Biomedical Research Center, Baton Rouge, LA, United States

**Keywords:** stress-related weight gain, human growth hormone, amino acid, somatostatin, fibromyalgia

## Abstract

**Introduction:**

An orally administered amino acid-based test supplement was recently shown to increase human growth hormone (hGH) in healthy adults. This prospective, observational, single-center, single-arm cohort study investigated the effects of 24 weeks of daily oral administration of the test supplement in individuals with stress-related weight gain, fibromyalgia (FM) and stress-related low-normal hGH production (15-30^th^ percentile for age-appropriate levels) on insulin-like growth factor 1 (IGF-1), an indicator of hGH levels caused by stress related stimulation of somatostatin.

**Methods:**

Participants continued to receive standard care. The primary endpoint was the change from baseline to endpoint (Week 24) in serum IGF-1. Additional endpoints included the change in body weight, clinical symptoms (assessed with the Revised Fibromyalgia Impact Questionnaire [FIQR], range 0-100, and Perceived Stress Scale [PSS], range 0-40), fasting cardiometabolic markers, tolerability, and safety. The study enrolled 84 fibromyalgia patients with low-normal age-adjusted IGF-1 serum levels. High mean ± Standard Deviation (SD) baseline FIQR and PSS scores of 76 ± 16 and 32 ± 5, respectively, indicated poor to moderate symptom management with standard care. All individuals completed 24 weeks.

**Results:**

Serum IGF-1 levels increased with a Week 24 mean± Standard Error (SE) change of 28.4 ± 3.0 ng/mL (*p*<0.001). Body weight was reduced with a Week 24 mean ± SE change of -5.5 ± 0.3 kg (*p*<0.001) (a 6.5% weight loss from baseline). The change from baseline in FIQR and PSS scores were -29.1 ± 1.1 and -20.0 ± 0.8, respectively (both *p*<0.001), indicating a substantial improvement. Statistically significant improvements from baseline to Week 24 were observed in systolic and diastolic blood pressure, HbA1c, LDL and HDL cholesterol, and triglycerides (all *p*<0.001). The supplement was well tolerated; no adverse events were reported.

**Discussion:**

Sustained augmentation of IGF-1 with the test supplement may represent a novel method of improving clinical symptoms, including stress-related weight gain, in individuals with fibromyalgia and stress-associated low-normal hGH.

## Introduction

Fibromyalgia (FM) is a syndrome characterized primarily by chronic pain, fatigue, many times with significant weight gain, and sleep disturbance that affects approximately 3-8% of the general population (1–3). It is most prevalent in women (1). Fibromyalgia is most commonly diagnosed in individuals aged 20-50 years but the incidence increases with age so that approximately 8% of adults meet classification criteria for fibromyalgia by age 80 (4). Other common concurrent symptoms include increased stress, disturbed sleep, impaired cognition, fatigue, mood disorders, other pain disorders, endocrine disorders, and obesity (1, 5, 6). There is no cure for fibromyalgia; treatment is individualized based on patient symptoms and may include medications, behavioral approaches, and lifestyle modifications, including regular exercise. The benefits of consistent resistance and aerobic physical activity regime, as well as multimodality physical therapy have been demonstrated in the current literature (7–10).

Several studies indicate that reduced production of human growth hormone (hGH) is evident in approximately 30% of individuals with fibromyalgia and may play a role in its pathophysiology (11–14). Impaired hGH production in these individuals is hypothesized to contribute to common fibromyalgia symptoms and comorbidities, such as fatigue, disordered sleep, impaired cognition, decreased lean body mass, increased adipose tissue, muscle weakness, and poor general health (11, 14). Furthermore, preliminary studies have demonstrated that treatment with rhGH produces an improvement in tender points and fatigue in individuals with fibromyalgia (15, 16). There are no Food and Drug Administration (FDA) approved treatments for increasing hGH levels in individuals with FM who do not have hGH deficiency (below the 15^th^ percentile for age). Thus, rhGH therapy in individuals with low hGH levels (but higher than the cutoff for GHD) is expensive, requires dose monitoring and adjustment, and may result in adverse effects. Additionally, the incidence of FM and low hGH increases with age (4, 17). However, treatment of fibromyalgia in elderly patients is complicated by greater prevalence of comorbidities and polypharmacy, as well as the increased risk of adverse drug reactions. There is a need for safe and effective methods to increase hGH in patients with fibromyalgia and low hGH, including the elderly.

An oral amino acid-based test supplement was recently shown to increase release of endogenous hGH in healthy individuals (18). The test supplement is hypothesized to enhance the release of hGH by suppressing somatostatin (19) and preliminary observations indicate that continued administration of the supplement promotes sustained improvements in hGH levels (19). This open-label, single-arm study investigated the effects of 24 weeks of daily oral administration of the amino acid-based test supplement in addition to standard care on serum IGF-1, a surrogate marker of hGH secretion in individuals with treatment-resistant fibromyalgia and low-normal hGH production (between the 15th and 50th percentile for age-appropriate levels of IGF-1) (13). Impaired hGH production can be identified by reduced levels of insulin-like growth factor (IGF-1), a mediator of hGH action and a long-term indicator of hGH levels (17). We hypothesized that oral administration of the supplement for 24 weeks would increase IGF-1 levels and improve clinical symptoms in individuals with treatment-resistant fibromyalgia and low-normal IGF-1.

## Materials and methods

### Study design

All studies were conducted in accordance with the principles of the Declaration of Helsinki and Good Clinical Practice. Written informed consent was obtained from all participants in all studies. The study is registered at clinicaltrials.gov, identifier: NCT04510181.

This was a prospective, open-label, single-arm, observational, single-center study designed to assess the efficacy of the test supplement in individuals with low-normal hGH production over 24 weeks. The supplement was well tolerated. No participants withdrew from the study early and no adverse events were reported. There were no adverse changes in vital signs, laboratory markers, or physical examinations.

Participants were recruited and 84 fibromyalgia patients were enrolled with low-normal age-adjusted IGF-1 serum levels from the Study Site (Private Practice of Susan Pekarovics, MD, Inc., Los Angeles, CA) between 2018 and 2020. All enrolled patients completed the study. Eligible participants were males and females between the age of 18 and 80 years and had a clinical diagnosis of fibromyalgia (according to the 2010 American College of Rheumatology criteria (6)) for which they were receiving standard care at the Private Practice of Susan Pekarovics, MD, as well as fibromyalgia-related comorbidities, including low-normal hGH production (between the 15^th^ and 50^th^ percentile for age-appropriate levels of IGF-1) (11, 12). Female participants of childbearing potential agreed to avoid pregnancy during the study. Participants were excluded from the study if they were diagnosed with human growth hormone deficiency (GHD), defined as IGF-1 levels below the 15th percentile for age appropriate levels) (11, 12), had previous treatment with rhGH, a history of substance abuse, or a history or presence of a severe mental illness, or at the discretion of the investigator.

After informed consent was obtained, demographics and baseline medical history were collected, and a baseline psychological examination was performed. Eligible participants returned for a baseline (Week 0 visit). Participants were instructed to self-administer the test supplement daily 30 minutes prior to bedtime on an empty stomach. The test supplement consists of 4 capsules containing 2.9 g of blended (listed in descending order) L-lysine, L-arginine, oxo-proline, N-acetyl-l-cysteine, L-glutamine, and *Schizonepeta tenuifolia*) and is commercially available (Basic Research, LLC). For participants who purchased the test supplement, the investigator verified that participants purchased the correct product. For participants who were financially limited and unable to purchase the amino acid-based blend, samples were provided by Sierra Research Group, LLC. No compensation was given for participation in the study.

All visits (Weeks 0, 6, 12, 18, and 24) were conducted in the morning and participants were instructed to be fasted (except for water) from 9:00pm the preceding evening. At each visit, compliance with the study protocol was assessed by the investigator by asking open ended questions about taking the test supplement, the assigned diet, an exercise program, blood draws were conducted for assessment of IGF-1, insulin-like growth factor binding protein-3 (IGFBP-3), fasting lipid panel (total cholesterol, LDL cholesterol, HDL cholesterol, triglycerides), fasting glucose, and HbA1c and participants completed the Revised Fibromyalgia Impact Questionnaire (FIQR) and Perceived Stress Scale (PSS). Adverse events were collected at each visit.

Before entering the study, participants were receiving standard care for fibromyalgia at the Private Practice of Susan Pekarovics, MD. Standard care for fibromyalgia consists of symptom management and primarily includes medication, physical therapy, dietary advice (28 kcal/kg/day), exercise advice (walk 45 minutes/day). Study procedures were conducted during standard care visits. Evaluation of symptoms was conducted at each visit and changes to standard care were made at any time during the study at the discretion of the Investigator. Participants could withdraw from the study at any time for any reason and receive standard care for fibromyalgia at the Private Practice of Susan Pekarovics, MD.

### Outcomes

The primary endpoint was the change from baseline (Week 0) to endpoint (Week 24) in serum IGF-1 (Quest Diagnostics, Los Angeles, CA). Additional endpoints included the change from baseline to Week 24 in body weight, and fibromyalgia symptoms (assessed with the FIQR) and stress symptoms (assessed with the PSS). The FIQR is a 21-item self-report measure that estimates the severity and impact of FM (20). Score ranges from 0-100 with higher scores indicating greater severity/impact of FM. The PSS is a 10-item self-report measure that assesses the perception of stress (21). Total score ranges from 0-40 with higher scores indicating greater perceived stress. Other outcomes included the change from baseline to Week 24 in IGFBP-3, body weight, systolic and diastolic blood pressures, HbA1c, and fasting lipids (Quest Diagnostics, Los Angeles, CA). Adverse events and safety were monitored by the Investigator who evaluated medical history, exploring the patients for any new complaints, and performed physical exams to detect any pathological changes, asked open-ended questions related to tolerability, and reviewed vital signs and laboratory markers.

### Statistical analysis

A paired t-test was used to compare changes from baseline to endpoint (Week 24). Data are presented as mean ± SD or mean ± SE. *p*<0.05 is considered statistically significant.

## Results

The study enrolled 84 participants (56 female, 28 male) and all completed 24 weeks of treatment. The mean ± SD baseline age was 67 ± 11 years. Consistent with entry criteria, mean ± SD serum IGF-1 was 107 ± 43 ng/mL. The relatively high mean ± SD baseline FIQR score of 76 ± 16 and PSS score of 32 ± 5 reflect poor to moderate control of fibromyalgia symptoms (20, 21). Baseline cardiometabolic markers indicate slightly elevated blood pressure, moderate dyslipidemia, and impaired fasting glucose (**Table 1**).

**Table 1:** Baseline and week 24 assessments for all participants.

	Baseline	Week 24	Change	Mean 95% CI
Body weight (kg)	85 ± 19	79 ± 19	-5.5 ± 0.3***	-6.1, -5.0
IGF-1 (ng/mL)	107 ± 43	135 ± 41	28.4 ± 3.0***	22.3, 34.6
IGFBP-3 (ng/mL)	3.8 ± 1.3	4.7 ± 6.9	0.9 ± 0.7	-0.5, 2.4
FIQR total score	76 ± 16	46 ± 16	-29.1 ± 1.1***	-31.2, -27.0
PSS total score	32 ± 5	12 ± 7	-20.0 ± 0.8***	-21.6, -18.5
Systolic blood pressure (mm Hg)	133 ± 11	113 ± 15	-20.6 ± 1.7***	-23.9, -17.2
Diastolic blood pressure (mm Hg)	84 ± 11	73 ± 7	-11.1 ± 1.2***	-13.4, -8.7
HbA1C (%)	7.0 ± 1.8	6.2 ± 1.1	-0.8 ± 0.1***	-0.9, -0.6
LDL cholesterol (mg/dL)	132 ± 45	101 ± 30	-32.2 ± 3.2***	-38.5, -25.9
HDL cholesterol (mg/dL)	52 ± 19	60 ± 19	8.1 ± 0.6***	6.9, 9.2
Triglycerides (mg/dL)	203 ± 124	129 ± 42	-73.7 ± 11.4***	-96.3, -51.1

Baseline and week 24 data are mean ± SD. Change data are mean ± SE. N=84. ***p<0.001 for comparison of baseline vs week 24 using a paired t-test.

**Figure 1:**
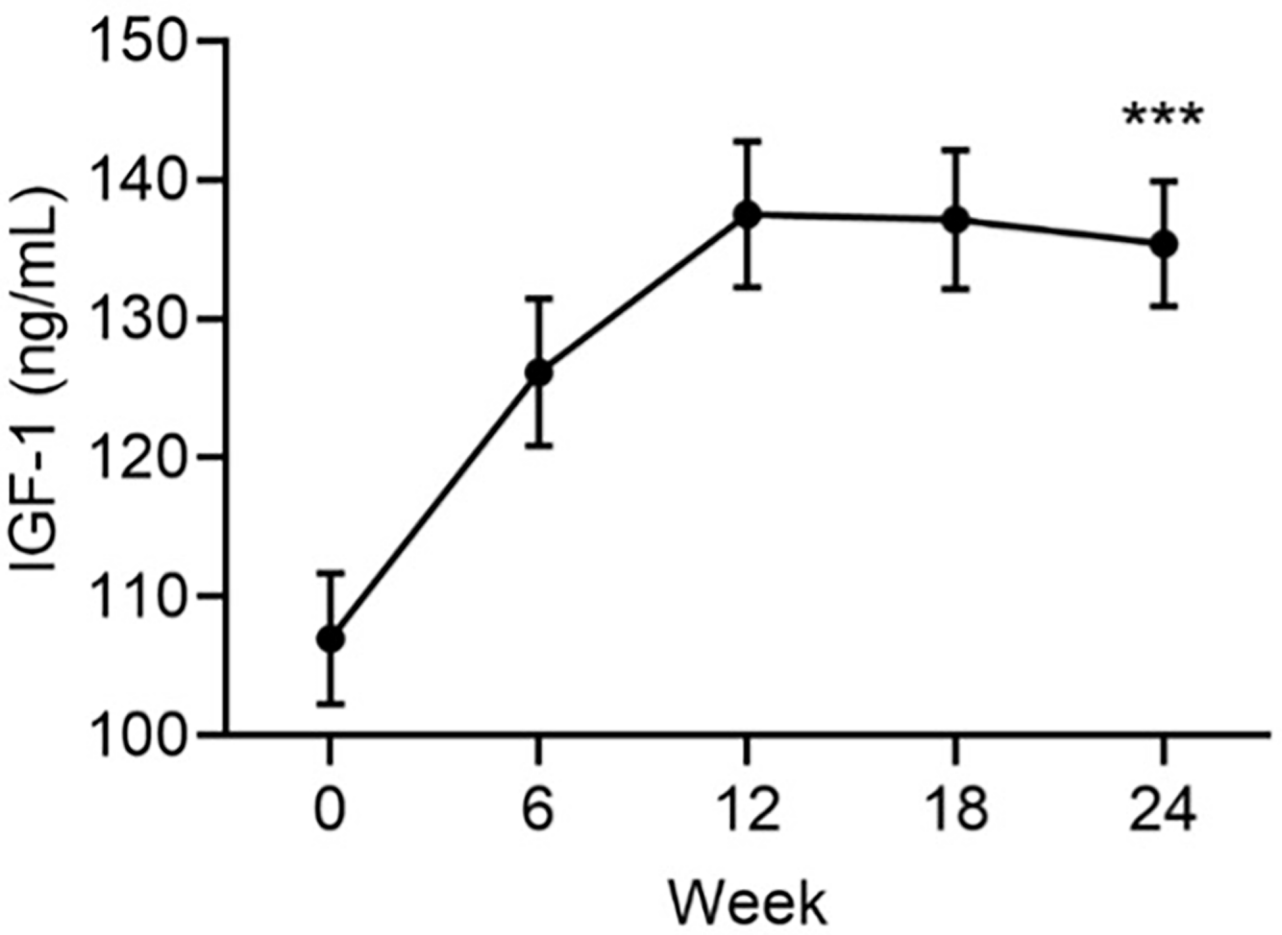
Mean serum levels of IGF-1 by visit. Data are mean ± SE by visit for all participants (N=84). ***p<0.001 for baseline vs endpoint.

There was an increase from baseline in mean IGF-1 levels that peaked at Week 12 remained elevated for the remainder of the study (**Figure 1**). At Week 24, the mean ± SE increase from baseline in serum IGF-1 was 28.4 ± 3.9 ng/mL (*p*<0.001). IGFBP-3 levels were unchanged from baseline (mean ± SE change: 0.9 ± 0.7, p=ns).

**Figure 2:**
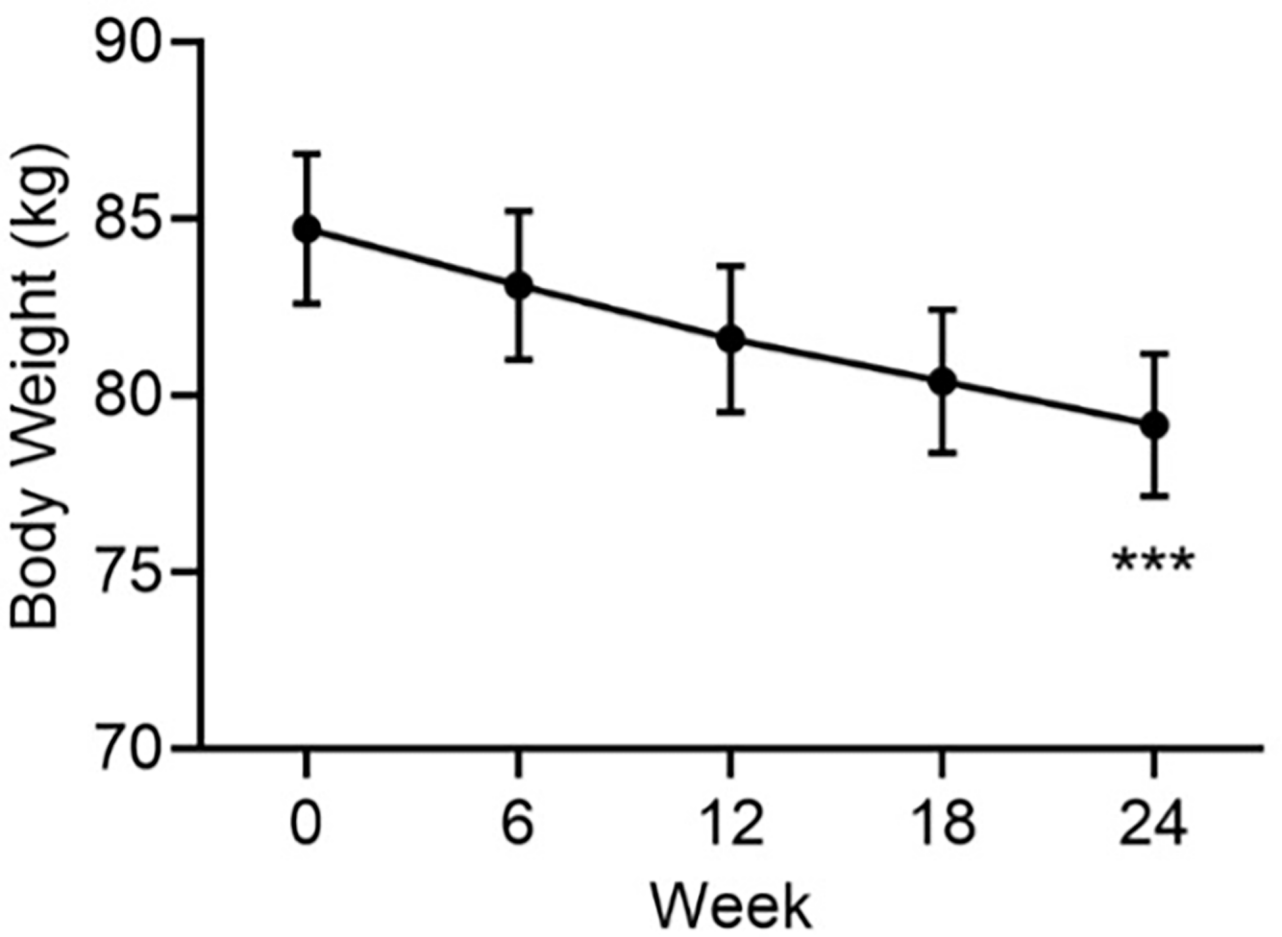
Mean body weight by visit. Data are mean ± SE by visit for all participants (N=84). ***p<0.001 for baseline vs endpoint.

There was a progressive reduction from baseline in mean body weight that continued for the duration of the study (**Figure 2**). At Week 24, the mean ± SE change from baseline in body weight was -5.5 ± 0.3 kg (*p*<0.001). This was a mean weight loss of 6.5%, a clinically significant weight reduction.

**Figure 3:**
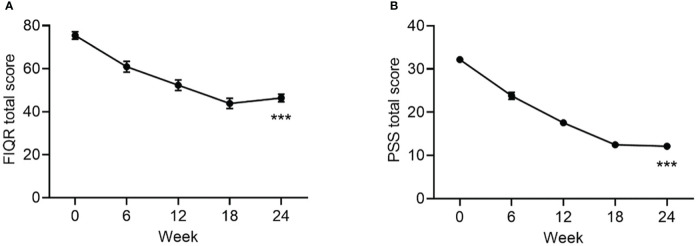
Change from baseline to Week 24 in FIQR **(A)** and PSS Scores. **(B)** Data are mean ± SE by visit for all participants (N=84). FIQR, Revised Fibromyalgia Impact Questionnaire; PSS, Perceived Stress Scale. ***p<0.001 for baseline vs endpoint.

The mean change in FIQR score from baseline to endpoint was statistically significant (**Table 1**, **Figure 3**). At baseline, the mean ± SD PSS score was 32 ± 5 and the mean ± SE change from baseline in PSS score was -20.0 ± 0.8 (*p*<0.001).

**Figure 4:**
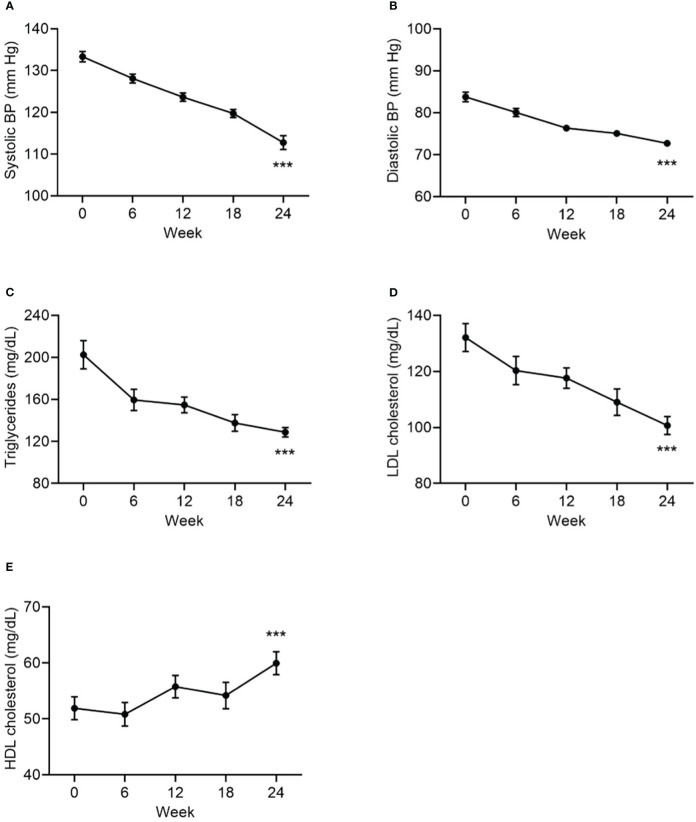
Change from baseline to Week 24 in systolic BP **(A)**, in diastolic BP **(B)**, in triglycerides **(C)**, in LDL cholesterol **(D)**, and HDL cholesterol **(E)**, as cardiometabolic markers. Data are mean ± SE by visit for all participants (N=84). BP, blood pressure. ***p<0.001 for baseline vs endpoint.

Statistically significant improvements from baseline to Week 24 were observed in all cardiometabolic measures: systolic and diastolic blood pressure, HbA1c, triglycerides, LDL-cholesterol, and HDL-cholesterol (**Table 1**, **Figure 4**).

The supplement was well tolerated. No participants withdrew from the study early and no adverse events were reported. There were no adverse changes in vital signs, laboratory markers, or physical examinations.

## Discussion

In individuals with suboptimal control of fibromyalgia and low-normal IGF-1 levels, the supplement resulted in an increase in IGF-1 levels from baseline. Additional improvements were observed in body weight, fibromyalgia and stress symptoms, blood pressure, A1C, and lipids. All individuals were receiving treatment for fibromyalgia that adhered to standard of care guidelines (1, 6). There was a 2:1 ratio of female to male participants, which is consistent with the incidence of fibromyalgia (1).

Human growth hormone (hGH) is primarily known for its vital role in the tissue and bone growth but also influences sleep, stress, pain, mood, and quality of life (1–3, 22, 23). hGH production and release from the pituitary is primarily stimulated by growth hormone releasing hormone (GHRH) and inhibited by somatostatin. The majority of hGH release occurs at the onset of slow wave sleep when GHRH levels are elevated and somatostatin tone is low (22, 23). hGH levels are highest during puberty and decline with age (24, 25). Low levels of hGH and IGF-1 are well documented in individuals with fibromyalgia (12, 13, 15, 26, 27). In the approximately one-third of patients with fibromyalgia and GHD, treatment with rhGH is appropriate and has been demonstrated to improve clinical symptoms (15, 26). In women with fibromyalgia and GHD, improvements in fibromyalgia symptoms and tender point scores were observed approximately 6 months after beginning treatment with rhGH (15). One study examined the effect of treatment with a low dose of rhGH on fibromyalgia outcomes in women with fibromyalgia and low to moderate hGH levels (defined as IGF-1 levels below 150 ng/mL at baseline) (16). Treatment with a low dose of rhGH for up to 12 months resulted in an increase in IGF-1 and improvement in fibromyalgia impact scores. In our study, which included a similar patient population and baseline IGF-1 and fibromyalgia impact scores as reported by Cuatrecasas et al. (16), an increase in IGF-1 was observed, as well as improvements in the FIQR and FIQR subscales. Thus, our results are consistent with previous beneficial reports of the effects of hGH replacement in individuals with fibromyalgia and low IGF-1.

Fibromyalgia is often comorbid with obesity and metabolic perturbations, such as insulin resistance and elevated leptin (28, 29). At baseline, the mean HbA1c of the population was 7.6%, indicating elevated fasting blood glucose. The population also exhibited dyslipidemia and slightly elevated blood pressure. After 24 weeks of administration of the supplement, there was weight loss of nearly 6 kg (approximately 1 kg per month), a reduction in mean HbA1c of 0.8%, and statistically significant improvements in systolic and diastolic blood pressure and lipids. The high baseline HbA1c levels and dyslipidemia observed in our study is consistent with the known inverse correlation between low IGF-1 levels and insulin resistance (30) and metabolic effects of IGF-1 (31). Furthermore, improvements in cardiometabolic parameters such as HbA1c, blood pressure, and lipids, is known to be associated with restoring IGF-1 levels to physiological levels in individuals with low IGF-1 (32). Although the cause of weight loss and improvement in cardiometabolic parameters requires further study, they are consistent with normalization of IGF-1 levels (31, 32), reduction in stress, and the known benefits of weight loss (33, 34) in this population.

Stress in among the most common psychological features of fibromyalgia. During chronic stress, elevated corticotropin-releasing hormone increases hypothalamic somatostatin tone (11, 23). Increased somatostatin tone has been observed in fibromyalgia and modulates the stress-induced suppression of pituitary hGH release (11, 12, 27, 35–39). The PSS reflects dynamic changes in stress (40). In general, individuals with fibromyalgia score higher on the PSS and reductions in PSS score are associated with an improvement in fibromyalgia symptoms and impact (21, 40, 41). This is consistent with the high baseline PSS score and reduction in PSS score that was observed in the study. Thus, our results indicate that there was an improvement in perceived stress over the course of the study. The reductions in perceived stress observed in this study may be attributed to the known effects of some amino acids to reduce somatostatin tone under certain conditions (23, 42–44).

Interpretation of the results of this study is limited by the lack of a placebo comparator group. However, our study is unique in that all the individuals were already receiving treatment for fibromyalgia by the study investigator at the study site prior to enrolling in the study. There were no changes to the standardized care upon the start of the study except for the addition of the test supplement to their existing treatment. Standard care consisted of office visits every 6 weeks. At these visits, blood draws were performed for IGF-1, IGFBP-3, fasting lipid panels, fasting glucose, and HbA1c and participants completed the FIQR and PSS questionnaires according to the study protocol. The patients also were asked about any adverse events, new symptoms, and the list of their medications. Next, they received a physical exam, including measuring their vital signs. At the end of each visit the patients were evaluated for their compliance with the supplement, their assigned diet and exercise. Care and symptom management continued as usual upon entering the study. Notably, exercise and dietary advice were already a key component of the standard care being provided to all the patients at the study site prior to entry in this study and these recommendations were not changed upon entering or during the study. Whether the increase in IGF-1 and other benefits observed in this study cohort are sustained beyond 24 weeks remains to be determined. Further randomized clinical trials would be necessary with larger samples and using a control group in order to obtain a more robust inference from the results.

Due to the complicated etiology and advanced age of many individuals with fibromyalgia, and weight gain there is a need for therapies for fibromyalgia and obesity that are both safe and effective as an additional part of a diverse treatment plan. In addition, considering some similar features between fibromyalgia and prolonged critical illnesses, long-COVID, and post-ICU syndrome, a coordinated multidisciplinary effort to share insights by the research and clinical community could be more successful in tackling the challenges of fibromyalgia therapy. The hGH enhancing effects of the test supplement represents a potential low-risk and cost-effective treatment to amplify endogenous hGH and improve clinical symptoms, benefitting individuals with low-normal hGH such as fibromyalgia, especially as this population includes elderly patients where the risk/benefit ratio is of substantial concern. Future studies should be conducted for repurposing sustained augmentation of IGF-1 with the supplement in weight control, and to assess its benefits in otherwise healthy individuals with obesity and low-normal hGH.

## Data availability statement

The original contributions presented in the study are included in the article/supplementary material. Further inquiries can be directed to the corresponding author.

## Ethics statement

The studies involving human participants were reviewed and approved by Argus IRB. The patients/participants provided their written informed consent to participate in this study.

## Author contributions

SP was the Primary Investigator of this study and the primary author of this manuscript. AB was contributing to collecting and organizing the data, analyzing the results, and composing the manuscript. CK was assisting with statistical analysis of the results. SB and AH were consulting on literature review and background information, study design, interpretation of the results, and composing the discussion part of this manuscript. All authors contributed to the article and approved the submitted version.
